# A Remarkable Extra-Uterine Case

**Published:** 1816-04

**Authors:** Josiah Bartlett

**Affiliations:** Charlestown.


*For the London Medical and Physical Journal.*

### *A remarkable Extra-Uterine Case;* by JOSIAH BARTLETT, M. D. of Charlestown.

IN October 1807, I was called upon to attend Mrs. S. M. aged twenty, who stated that she had passed the common period of pregnancy with the usual symptoms, and that for several days she had felt what she was told were indications of labour. I visited her occasionally, in expectation of that event, two or three weeks, at which time her size lessened and her symptoms abated. Thus circumstanced, Dr. John Warren visited her in consultation, when it was agreed, to recommend patience, attend to the alimentary canal, and observe the operations of nature. All the appearances of parturition subsided, and she resumed the cares of her family. I frequently saw her until May 1808, when a hard substance was distinctly felt on the right side of the abdomen, a little below the umbilicus, which frequently produced uneasy sensations, especially during menstruation, and continued till the operation which is described in the sequel.

In October 1809, after a natural and easy labour, she was delivered of a healthy full grown child, and recovered without any uncommon symptoms. In November 1812, the same event occurred, and she did as well as before. In February 1815, she was again delivered of a healthy child, and for two weeks was as comfortable as in her former confinements. In each of these labours, I enquired if she suffered any peculiar pain or inconvenience from the tumour in her side, and she always answered in the negative.

March 1st. About this period she became feverish, with a loss of appetite, a diminution of strength, frequent sickness at the stomach and occasional rigours, but had no cough. I visited her every second or third day until the middle of May, during which time, the child was put out to nurse; she took several doses of ipecacuanha, such mild laxatives as regulated the bowels, and various tonic medicines, of which cinchona officinalis was the principal, but without any material change in her situation. She then applied to Dr. Marshall Spring, to whom I had no opportunity to relate the case; but it was stated that he attributed her "great weakness and loss of flesh to the swelling in her side." He prescribed the tincture of sanguinaria canadensis, and ipecacuanha occasionally, as the most suitable emetic, and the powder of cinchona officinalis with conserve of roses, in preference to its being taken in wine, as it had been administered.

June

June 21st. She again applied to me, and stated, that she had gained nothing by changing the form of her medicines, that she was weaker and greatly emaciated, though she was better able to take animal food, wine, &c. than when I last visited her. The bowels were in a good state, but the tumour was more troublesome, and there was a small red spot near the navel. I advised her to suspend the use of medicine, for a few days, and to depend on nourishment.

24th. In the evening, a large quantity of matter, which was so offensive that the family was obliged to leave the room, was discharged from the tumour. On the following morning I made a particular examination; the discharge, which had been copious through the night, was more fœtid than any thing I had ever noticed; the opening was but just sufficient to admit a probe, and I distinctly felt bones, about an inch and a half from the surface. In the afternoon she was visited by Dr. Thomas Welsh, and the next morning by Dr. John C. Warren, both of whom confirmed my opinion of feeling the bones.

27th. A consultation was held with the above named gentlemen, Drs. David Townsend, Abraham R. Thompson, and William J. Walker, when it was unanimously agreed, that the only method by which the patient could obtain relief, would be an attempt to remove the remains of a fœtus, which we supposed was situated in a sack, formed by an enlargement of the right fallopian tube adhering to the peritonæum. I resorted to this hazardous and uncertain remedy in their presence, and that of three medical students, (Messrs. Gorham Bartlett, John C. Dalton, and Josiah S. Hurd) by enlarging the orifice, to introduce my left fore finger, which, serving as a director, enabled me to dilate the wound, in the course of the linea alba, to such a size as to extract the child, the body of which had been full grown, and seemed to be doubled. The pelvis, (which was first extracted) spine, and ribs, were not disconnected, and there remained a portion of the integuments and viscera, which were too rotten and offensive to dissect; the bones of the head followed in different portions, which with those of the extremities, were removed by repeatedly introducing my hand. The hæmorrhage, during the operation (which the patient bore with great fortitude) was small, and the wound was dressed superficially, without an attempt to unite its edges. Light nourishment was directed, she passed the day comfortably, and at evening took a dose of tinctura opii.

28th. She had a good night. The discharge from the wound, which was dressed twice, was copious, very offensive and tinged with blood. She had a small evacuation of urine urine in the forenoon, and was able to lay on both sides, which had not been the case for a long period. Her diet was light and nutritive; the tinctura opii was directed to be taken in the evening, if necessary, with orders to administer a dose of calcined magnesia early in the morning.

29th. She took the magnesia about twelve o'clock, and passed a comfortable night without the anodyne. At six, A. M. I received information that the medicine had passed by the wound. On visiting her, I found that the discharge was greater, and as offensive as ever, and I feared that it was in part from the intestines. Directed a strong decoction, and the compound tincture of cinchona officinalis to be taken as freely as she could bear. At three, P. M. there was a sudden and copious discharge from the wound, which was similar to that in the morning. Having renewed the dressings, I directed an injection; before evening she had four evacuations from the bowels, in which the magnesia was noticed, and she discharged urine freely, though accompanied with a bearing down, which she said resembled labour pains. She bore the medicine and nourishment, which consisted of wine-whey, punch, broth, &c. and was directed to take an anodyne if she was restless. Her pulse and countenance greatly improved, she was moved without difficulty, and repeatedly stood on her feet.

30th. She took the anodyne in consequence of another discharge from the bowels, and passed a good night. The wound was dressed twice, the discharge as yesterday, but less in quantity; she had evacuations from the bowels and bladder, took the cinchona, wine, &c. regularly, ate moderately of animal food, and passed a good day.

July 1st. She took an anodyne the last evening; her bowels in good order. The matter from the wound, which was dressed twice, less in quantity and much better. At evening she sat up half an hour. The medicine,. &c. continued.

2d. She had two evacuations from the bowels; the bearing down, at the discharge of urine, lessened; the appearance and discharge of the wound much improved; dressed morning and evening; she sat up twice this day, and continued the prescriptions.

3d. She took an anodyne last evening, and appeared rather more feeble than yesterday. The state of the wound improving, and the discharge much lessened. She had as usual two alvine evacuations, took animal food more freely, and increased the quantity of cinchona and wine.

4th. She passed the last night and this day very comfortably; the bowels in a good state; the pressing with the discharge of urine subsided; the digestion from the wound good, and it is gradually closing.

5th. The discharge from the wound was so small, and the appearance so favourable, that she required but one dressing. Her strength gaining rapidly under the treatment which was at first adopted, she was able to sit up two or three hours.

I continued to dress the wound daily until the 20th, when it was dressed every second or third day; the quantity of matter being very small. From this period the wound began to cicatrize, and at the date of this communication is so nearly healed, as only to discharge a small quantity of lymph. The patient has rode, visited her neighbours, and is in better health, than during the last eight years.

It should have been noticed, that I frequently injected warm water into the wound, and used alcohol freely over the dressings, which had a good effect in sweetening and strengthening the parts. The gentlemen who attended the operation, and others, called occasionally to notice the progress of the case, and I was much obliged by their attentions.

In the American Magazine, published at Boston in 1746, is related the history of a woman, with an extra uterine fœtus from 1731 to 1745, during which period she had six full grown children. In three weeks from her last parturition, there was an opening in the tumour near the navel, from which matter was discharged (about eight ounces in a day) for three months, when a portion of the head of a child was discovered, and some small bones were extracted; an incision was then made and the rest of the bones were removed at different periods, in four days, when the wound was stitched up. She died soon after; and, on dissection, it was ascertained that the fœtus had laid in the left fallopian tube, which was greatly distended, and adhered to the peritonæum; the right tube and the uterus were in a healthy state.

In the Massachusett's Medical Communications, Vol. I. Part III. p. 30, is an interesting account of an extra uterine case, which continued more than seventeen years, during which time the woman had four children and several abortions. She died in 1802, when it appeared on examination, that the "uterus and right fallopian tube were in a sound state; the left tube was greatly enlarged, and from it was taken the bones of a full grown fœtus."

These, with the exception of one in 1783, published in the Memoirs of the American Academy of Arts and Sciences, Vol. I. p. 551, where a quantity of hair was taken from an abscess in the abdomen, and a question arose relating to an extra uterine fœtus, are the only cases in our Commonwealth

 which which have come to my knowledge, that bear any resemblance to the one I have now related.

It is remarked by a distinguished writer on obstetrics, that "in the records of medicine there is a very great number of examples of the extra-uterine fœtus, in all of which there may be observed some similarity of circumstances;" that but "few practical remarks have been made on the subject, which can be useful to those who are in the way of meeting with cases of this kind. If an abscess should be formed in the side, or any part of the abdomen, and through the subsequent opening any part of the child should be evacuated, it will then be expedient to forward the exclusion of the remaining parts, either by enlarging the opening, or giving such other assistance, as surgery is very competent to afford."

It is further stated, that "when all the bones" of an extra-uterine fœtus "are evacuated by the intestines or vagina, the parts affected gradually recover from the injury they have sustained, without any remaining mischief, and the patient usually enjoys as perfect health, as if no such accident had happened."

Having examined all the books in my possession, I can find but a single instance where a putrid fœtus was extracted from an abscess in the umbilical region, and the mother was preserved: this is related in Heister's Surgery, Vol. II. Chap. CXIII. "on the cæsarean section."—*New-England Journal.*

